# The Role of Targeted Therapy and Immunotherapy in Metastatic GNET/Clear Cell Sarcoma (CCS) of the Gastrointestinal Tract: A Case Report

**DOI:** 10.3390/cimb47090706

**Published:** 2025-09-01

**Authors:** Raluca Ioana Mihaila, Andreea Veronica Lazescu, Daniela Luminița Zob, Dana Lucia Stanculeanu

**Affiliations:** 1 Department of Oncology, “Carol Davila” University of Medicine and Pharmacy, 050474 Bucharest, Romania; 2 Department of Medical Oncology I, “Prof. Dr. Alexandru Trestioreanu”, Institute of Oncology, 022328 Bucharest, Romania; 3 Department of Medical Oncology II, “Prof. Dr. Alexandru Trestioreanu”, Institute of Oncology, 022328 Bucharest, Romania

**Keywords:** gastrointestinal neuroectodermal tumour, GNET, clear cell sarcoma of gastrointestinal tract, CCS, clear cell sarcoma-like tumour of gastrointestinal tract, EWSR1 gene, EWSR1/CREB1, aggressive tumours, very rare disease, multidisciplinary approach, immunotherapy, targeted therapy

## Abstract

**Background**: Gastrointestinal neuroectodermal tumour (GNET), also known as clear cell sarcoma (CCS) of the gastrointestinal tract, is a rare neural crest-derived malignancy characterized by EWSR1-ATF1 or EWSR1-CREB1 fusions. Due to its rarity, there is limited evidence and no established guidelines for standard management. GNET is aggressive, with high rates of local recurrence, metastasis, and mortality. **Case Presentation**: We report the case of a 46-year-old woman with a family history of gastrointestinal cancers who was diagnosed in 2020 with an intestinal GNET. She underwent a segmental enterectomy as the first step of multimodal therapy. After three years of follow-up, she developed hepatic and peritoneal metastases. In November 2023, she began combined therapy with the anti-VEGF tyrosine kinase inhibitor cabozantinib and the immune checkpoint inhibitor nivolumab. The patient has maintained stable disease for 18 months with good tolerance and no adverse events. Molecular analysis of the tumour, which showed an EWSR1-CREB1 fusion, supported the selection of targeted therapy and immunotherapy as the preferred treatment approach. **Conclusions**: Immunotherapy and targeted therapy show promise for GNET/CCS treatment, but clinical standards are lacking, and evidence comes primarily from case reports. Additional data are needed to determine the best sequence and combination of therapies for this very rare disease.

## 1. Introduction

Malignant gastrointestinal neuroectodermal tumour (GNET), also referred to as clear cell sarcoma (CCS) of the gastrointestinal tract, is a rare and aggressive malignancy accounting for less than 1% of gastrointestinal sarcomas [1,2]. These tumours typically arise in the small intestine and present with nonspecific symptoms such as abdominal pain, gastrointestinal bleeding, altered bowel habits, or weight loss. Prognosis is poor due to a high risk of recurrence and metastasis, and evidence guiding systemic therapy remains limited [3,4,5].

Diagnosis relies on histopathology, immunohistochemistry, and molecular testing. GNET usually demonstrates a primitive neural phenotype, with expression of S-100, SOX10, synaptophysin, and CD56, while lacking melanocytic, GIST, epithelial, and myoid markers [2]. Molecular confirmation is often required, as most cases harbour EWSR1-ATF1 or EWSR1-CREB1 fusions [3,6]. Next-generation sequencing (NGS) therefore plays a critical role in establishing the diagnosis and identifying potential therapeutic targets.

Surgery with negative margins (R0) remains the cornerstone of treatment [7,8,9,10], and adjuvant radiotherapy may be considered in selected high-risk cases [11,12,13]. Chemotherapy regimens are usually adapted from soft tissue sarcomas, but their efficacy is modest, and no standardized protocol exists [14,15,16]. Given the limited benefit of conventional strategies, increasing attention has turned to molecularly guided approaches.

Targeted therapies, such as multi-tyrosine kinase inhibitors (cabozantinib, pazopanib, sunitinib), have shown clinical activity in individual cases [12,16]. Immunotherapy, particularly PD-1 blockade, has also demonstrated encouraging results in selected patients and recent sarcoma trials [17,18]. Case reports suggest that combining cabozantinib with immunotherapy may have synergistic effects by modifying the tumour microenvironment and enhancing T-cell activity [19].

Here, we present a case of relapsed GNET/CCS with EWSR1-CREB1 fusion, managed with combined nivolumab and cabozantinib. The patient achieved durable progression-free survival without toxicity, highlighting the potential of personalized, molecularly guided strategies in this ultra-rare malignancy.

## 2. Case Presentation

The patient, a 46-year-old woman with a significant family history of gastrointestinal malignancy (both parents died of colon cancer), underwent laparoscopic cholecystectomy in 2014. In June 2020, she was diagnosed with high intestinal obstruction caused by severe stenosis at the level of a jejunal loop. The symptoms had a sudden onset, beginning with bile-stained, food-containing vomiting—predominantly at night—followed by colicky abdominal pain recurring every 3–4 days. These episodes were accompanied by frequent belching, persistent hiccups, early satiety, diffuse abdominal gurgling (particularly in the upper abdominal region), and progressive weight loss of approximately 12 kg over the preceding month.

**Figure 1:**
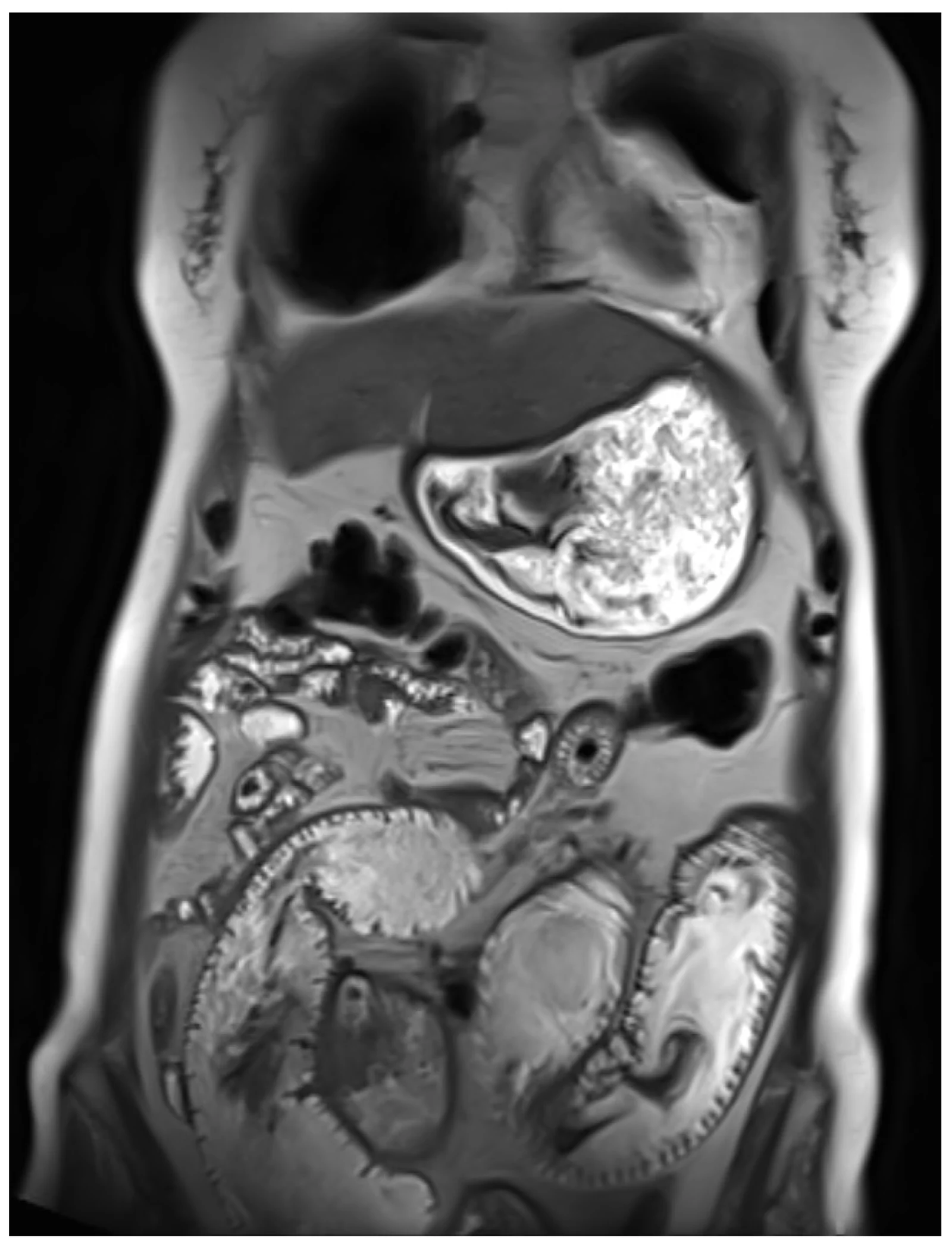
Dilation of intestinal loops on MRI.

The patient underwent multiple gastroenterological evaluations at different healthcare centres for progressive signs of intestinal obstruction. An entero-MRI was performed, revealing a circumferential, mildly asymmetric, and relatively short segment of thickening at a jejunal loop. This lesion caused significant upstream dilation of the proximal jejunal loops, with a maximal diameter of 5 cm—findings consistent with high-grade intestinal obstruction due to a tight stricture at the jejunal level. No local or distant metastases were identified at that time (Figure 1).

The patient was admitted to the surgical department with an acceptable performance status but presented with recurrent fever and abdominal pain. Clinical examination revealed abdominal hypertonicity on percussion, particularly in the upper abdominal region. There was no evidence of shifting dullness in the flanks. On deep palpation, mild tenderness was noted in the epigastric area, without signs of peritoneal irritation. Laboratory investigations on admission showed a mild, correctable electrolyte imbalance. A chest X-ray was also performed, which did not reveal any acute pleuro-pulmonary pathology. Given the clinical and imaging findings consistent with intestinal obstruction, surgical intervention was undertaken. A segmental enterectomy was performed, including isolation of the vascular supply to the affected jejunal loop, followed by a mechanical latero-lateral entero–entero anastomosis. The postoperative course was uneventful, with no complications reported.

Histopathological examination of the surgical specimen revealed a transmural tumour infiltrating the small intestine wall. The tumour was composed of relatively monomorphic epithelioid cells and, focally, spindle-shaped cells with vesicular nuclei, prominent nucleoli, and eosinophilic cytoplasm. The neoplastic cells were arranged in nests and compact islets, forming a network-like architecture. The lesion exhibited mucosal ulceration and invaded through all layers of the intestinal wall, reaching the subserosa. The tumour measured 2 cm in greatest dimension, and no metastatic involvement was identified in the excised lymph nodes. Immunohistochemical findings—summarized in Table 1—were consistent with a diagnosis of malignant gastrointestinal neuroectodermal tumour. The histological appearance and IHC profile were independently reviewed at the Victor Babeș National Institute and Medcenter in Bucharest, as well as at MD Anderson Cancer Centre in Spain, all of which confirmed the diagnosis.

**Figure 2:**
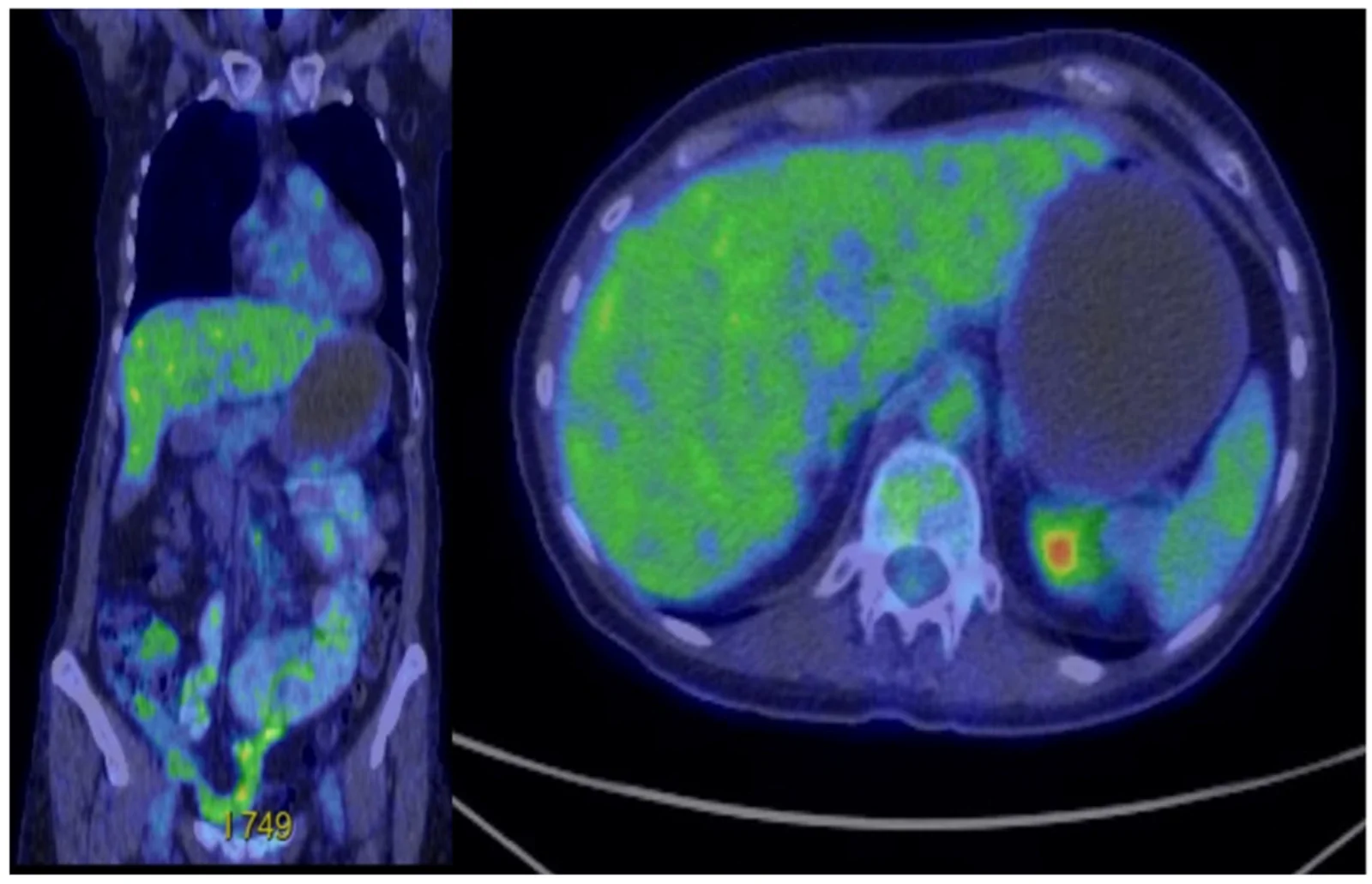
Postoperative PET-CT-no residual disease.

The patient was referred to our department for oncological evaluation. Given the rarity of the diagnosis and the limited data available in the literature regarding standardized adjuvant treatment protocols for GNET, a comprehensive assessment was initiated. We recommended a PET-CT scan to determine the extent of disease and assess metabolic activity, alongside repeat immunohistochemistry and molecular profiling through NGS or a Foundation One^®^ panel. The patient was also referred to an international sarcoma/rare disease institute for a second opinion. The PET-CT scan revealed no evidence of metabolically active disease (see Figure 2). Molecular analysis via NGS/Foundation One^®^ did not identify any actionable mutations. The tumour mutation burden (TMB) was low, microsatellite status was stable (MSS), and PD-L1 expression was negative (described in Table 2).

Based on the available literature—which indicated no significant benefit from adjuvant therapy in similar cases—and multiple second opinions from specialized centres (MD Anderson, Acibadem, and AKH Vienna), our multidisciplinary team concurred with a surveillance-based approach. The patient was therefore recommended for active follow-up, which included MRI scans (consistent with the baseline diagnosis via entero-MRI), routine blood tests, and clinical examinations at intervals of 3–4 months. Between November 2020 and February 2023, the patient remained under surveillance in our department, with no clinical or imaging evidence of disease progression during this period.

In February 2023, abdominal and pelvic MRI re-evaluation showed no signs of local tumour recurrence at the site of the entero–entero anastomosis. However, it revealed a 2 cm nodular hepatic lesion located at the junction of segments IVa and VIII, along with two additional smaller nodular liver lesions—one at the junction of segments VI and VII, and another within segment VIII. The imaging appearance of these lesions was inconclusive, and further evaluation was recommended, including PET-CT to assess metabolic activity and exclude tumour recurrence, liver MRI for detailed characterization, and a surgical consultation. At follow-up in our clinic, the patient maintained an excellent performance status, with normal laboratory results. Based on these findings, we recommended a liver MRI, PET-CT, and referral for surgical or interventional radiology consultation to consider biopsy and confirm possible disease progression. Subsequent PET-CT confirmed disease relapse, revealing metabolically active perihepatic peritoneal lesions—measuring up to 10 mm near segment IVa/VIII, with additional smaller lesions around segments VIII and VI–VII (with an SUVmax raging between 11–13).

Following surgical consultation, the patient underwent an atypical hepatectomy of segment IVa in May 2023, along with a biopsy of a peritoneal nodule. The procedure was performed for diagnostic purposes and appeared to achieve macroscopic clearance (R1 resection). Final histopathological and immunohistochemical analysis confirmed liver and peritoneal metastases of GNET/CCS, with PD-L1 expression showing a tumour proportion score (TPS) of 15%. Unfortunately, a postoperative MRI revealed two liver metastases showing dimensional progression. A second NGS analysis performed on the liver metastasis did not reveal any additional actionable genetic alterations for treatment selection. The tumour remained microsatellite stable (MSS), with a tumour mutational burden (TMB) of 4 mutations/Mb and confirmed the presence of the EWSR1-CREB1 fusion. PD-L1 expression remained at 15% TPS. The discrepancy of the initial and subsequent molecular tests may reflect tumour evolution over time or technical variability in molecular testing and highlights the dynamic biology of these ultra-rare malignancies. Subsequent PET-CT confirmed progression, with active peritoneal and liver lesions (the hepatic lesion had an SUVmax of 12.4, and the largest peritoneal nodule an SUV max of 11.5). The patient then sought further follow-up and management at another clinical centre, with recommended cytoreductive surgery followed by a combined regimen of immunotherapy and targeted therapy—specifically, nivolumab and cabozantinib. Chemotherapy was considered a secondary option, to be pursued only if the patient was unable to access first-line targeted treatment due to lack of reimbursement by the National Health Insurance Program. Literature data suggest that GNET/CCS cases may respond to various targeted agents, including MET kinase inhibitors (such as crizotinib, cabozantinib, or tivantinib), VEGFR inhibitors (such as pazopanib or sunitinib), and immune checkpoint inhibitors. The therapeutic options we proposed were based on an individualized approach, incorporating the patient’s molecular and tumour-specific characteristics. These included PD-L1 expression of 15% (TPS), presence of the EWSR1-CREB1 fusion, absence of other actionable oncogenic drivers, and a Ki-67 index of 30%. Our decision was guided by case-based evidence from the literature, with the rationale that cabozantinib, a multi-kinase inhibitor, may provide a clinically meaningful response, as well as that co-administration with a PD-1 inhibitor (nivolumab) could counteract T-cell exhaustion, potentially producing a synergistic antitumor effect [19,20]. Due to rapid progression of liver and peritoneal metastases, with minimal response to prior local therapies and a high risk of further disease advancement, achieving a rapid therapeutic response was deemed essential.

In December 2023, the patient underwent surgical cytoreduction at another clinical centre. The complex procedure included omentectomy, peritonectomy, liver metastasectomy, jejunal resection, and right hemicolectomy. The surgery was completed without any postprocedural complications. Histopathological and immunohistochemical analysis confirmed the presence of GNET/clear cell sarcoma CCS metastases in the resected peritoneal, jejunal, and colonic tissues. However, the liver lesions were negative for malignancy. Molecular profiling (NGS panel) of the cytoreductive surgery specimens identified PD-L1 expression with a tumour proportion score (TPS) of 15%. Variants of unknown significance (VUS) were detected in the MYC, EWSR1, and STAG2 genes. Based on the molecular findings and current literature, cabozantinib was recommended as a targeted therapeutic option according to the report.

**Figure 3:**
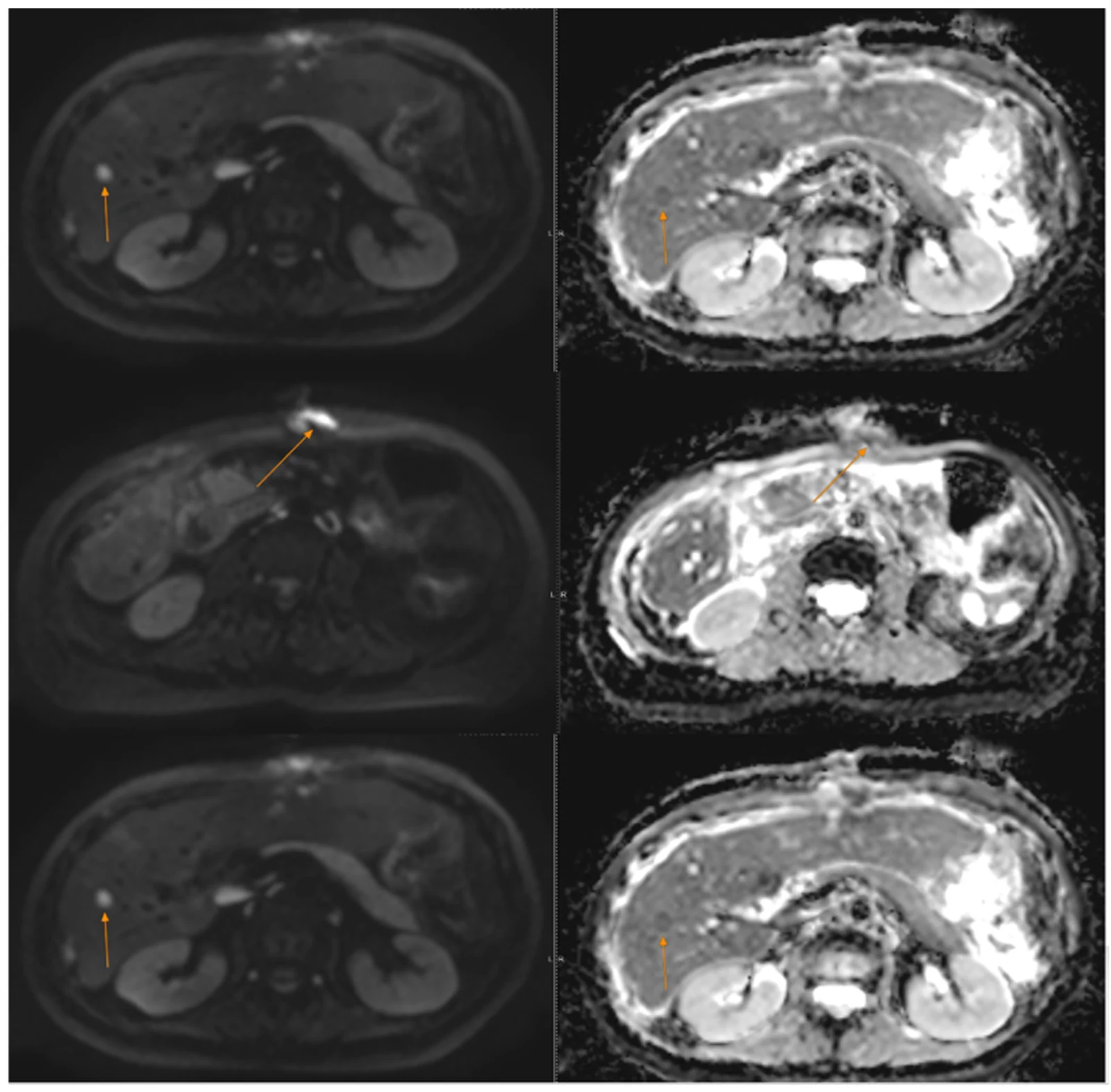
Liver and peritoneal metastases on MRI post-cito-reductive surgery.

At the time of consultation in our department, the patient’s general health status and biological parameters were excellent. Although the surgery had cytoreductive intent, the postoperative MRI detected residual disease (peri centimetric peritoneal and hepatic metastases)—confirmed by postoperative MRI (see Figure 3). T2W heterogeneous lesions displaying restricted diffusion are observed. These lesions measure 18 × 14 mm on peritoneal surface at the level of liver segments 8–4, 22 × 8 mm and 25 × 10 mm adjacent to each other at the level of segment 2 and 20 × 9 mm adjacent to surgical site on peritoneal surface at the level of segment 6. The patient had a good performance status, no comorbidities, and no contraindications to combined immune-targeted therapy. After multidisciplinary discussion, we opted for treatment with a MET kinase inhibitor (cabozantinib) in combination with a PD-1 checkpoint inhibitor, based on its more favourable safety profile compared to other VEGFR inhibitors such as pazopanib or sunitinib [21]. Furthermore, the NGS panel from the cytoreductive surgery identified PD-L1 expression at 15% TPS, with variants of unknown significance (VUS) in MYC, EWSR1, and STAG2. Given the patient’s prior intolerance and lack of benefit from chemotherapy, first-line targeted therapy combined with immunotherapy was considered a rational and potentially more effective therapeutic option. The combination of cabozantinib, a multi-target tyrosine kinase inhibitor with activity against MET, VEGFR, and AXL, and nivolumab, an immune checkpoint inhibitor targeting PD-1, was selected based on their potential synergistic effect. Cabozantinib may enhance antitumor immunity by modifying the tumour microenvironment, reducing immunosuppressive signalling, and promoting T-cell infiltration, thereby potentiating the efficacy of PD-1 blockade. Although prospective evidence in GNET/CCS is lacking, emerging data in other sarcomas and rare tumours support this strategy as a rational approach in the absence of standard treatment options.

**Figure 4:**
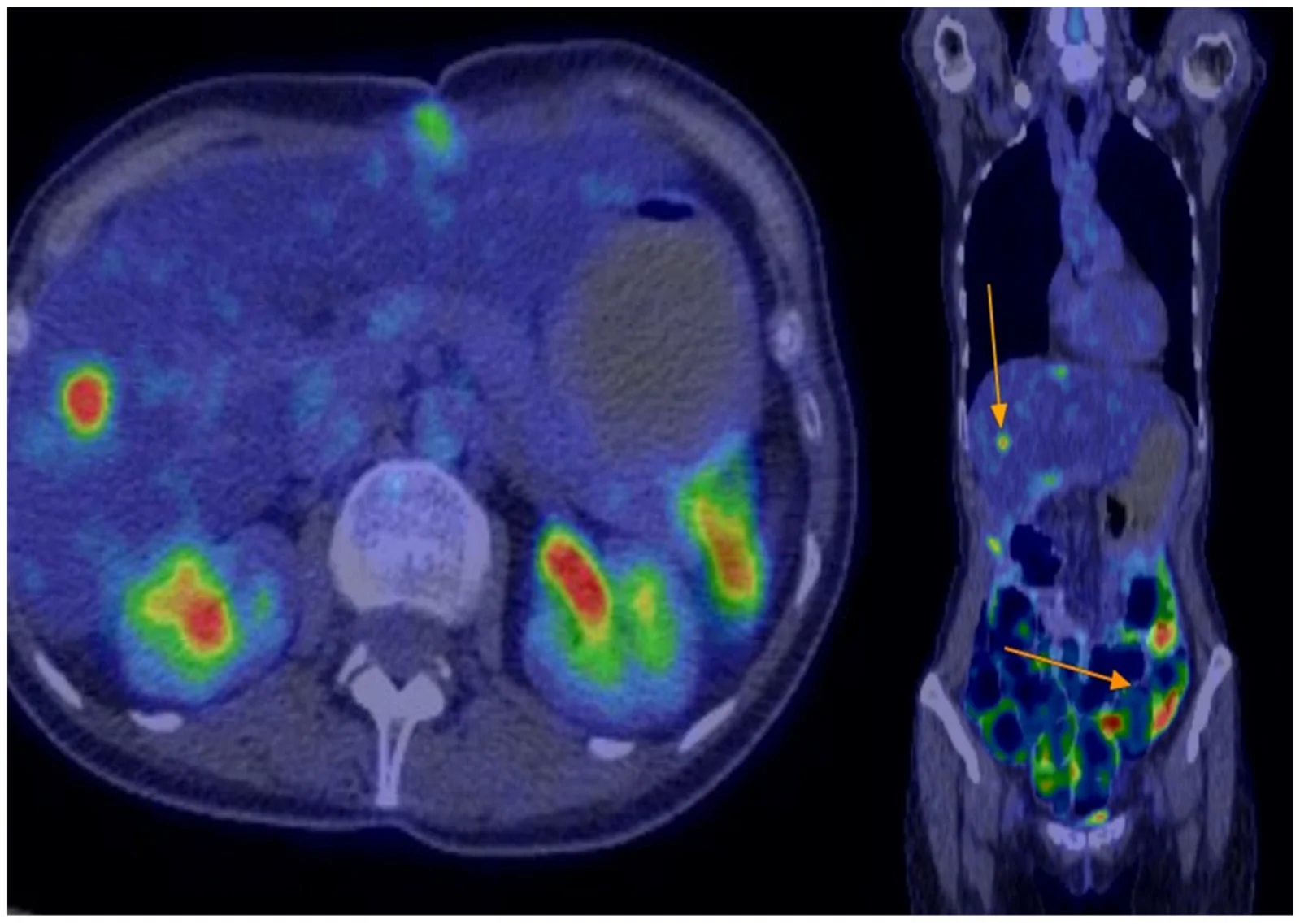
Treatment response on PET-CT evaluation at 9 months.

Treatment was initiated with nivolumab 480 mg every 4 weeks and cabozantinib 40 mg daily. This regimen was maintained for the first three months, with scheduled imaging assessments—including PET-CT and hepatic MRI—at 3 to 4 months to evaluate both structural changes and metabolic activity of liver and residual peritoneal metastases. Monthly clinical and laboratory follow-up was performed, during which the patient showed good treatment tolerance without adverse events. Follow-up imaging at 3 and 6 months demonstrated no disease progression on PET-CT or hepatic MRI. At the 9-month evaluation, liver MRI identified three new small lesions (measuring 5–8 mm). Two of these showed slight dimensional progression, while one showed regression compared to the previous scan. According to RECIST 1.1 criteria, the overall assessment remained consistent with stable disease. To further evaluate metabolic activity, an additional PET-CT was performed (see Figure 4). It revealed multiple nodular and flat lesions located along the anterior peritoneal surface of segments III, IV, VI, and VIII (SUV max up to 6.66; largest lesion 5.3 × 1.3 cm), as well as intraparenchymal lesions in segment V (SUV max 4.62) and segment VI (1.5 × 1.3 cm, SUV max 9.22). On PET-CT prior to treatment, the hepatic lesion had an SUVmax of 12.4, which decreased to 5.2 at 6 months and remained stable at 18 months. The maximum SUV of the largest peritoneal nodule decreased from 11.5 to 7.3 cm. These findings supported the clinical observation of durable disease control. Despite these findings, the disease remained classified as stable.

Treatment was continued, both clinical and standard biological evaluation, before each cycle; imaging follow-up was scheduled at three-month intervals. The most recent evaluation, conducted in June 2025, showed no evidence of tumour recurrence at the site of the entero–entero anastomosis. MRI imaging revealed discrete dimensional progression in two hepatic lesions (from 5 × 5 mm to 8 × 7 mm, and from 8 × 7 mm to 10 × 8 mm), while a third lesion demonstrated a slight reduction in size (from 8 × 8 mm to 6.5 × 8 mm). Overall, the MRI findings were consistent with stable disease, as defined by RECIST 1.1 criteria. The patient continues the current treatment regimen, with regular follow-up and no reported adverse events (according to CTCAE criteria). As of now, the patient has achieved an 18-month period of progression-free survival.

**Figure 5:**
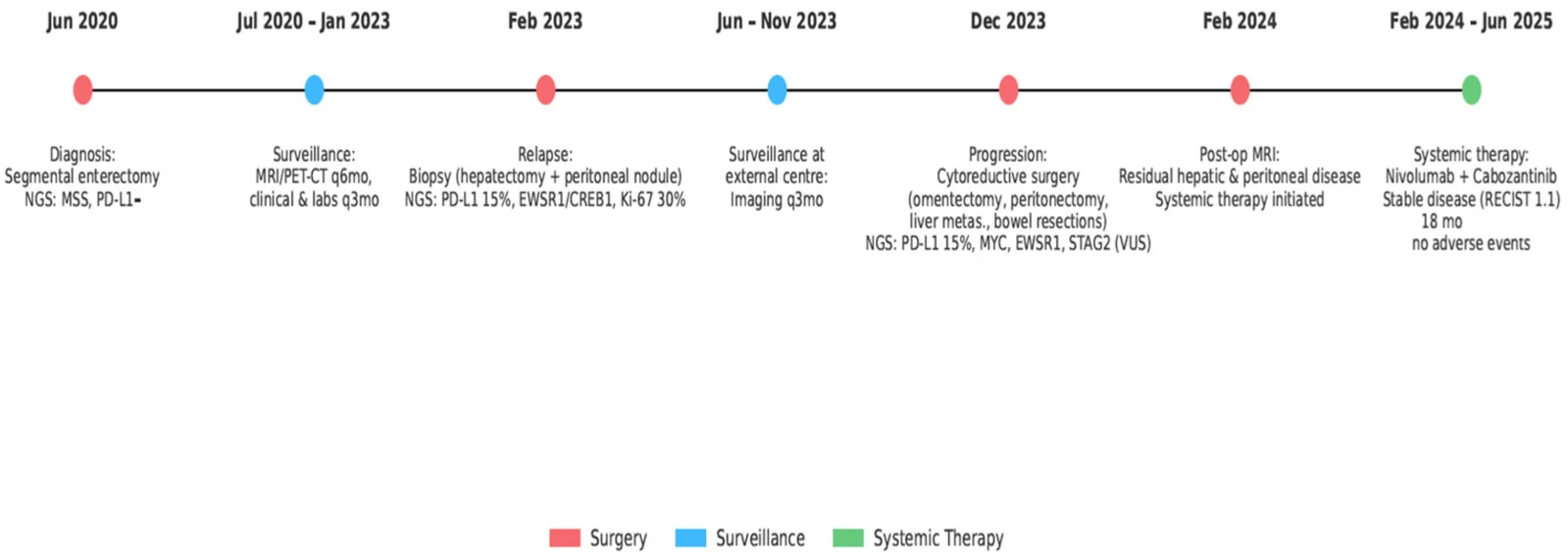
Patient’s diagnosis and treatment timeline.

Key milestones in the patient’s diagnostic and therapeutic journey are summarized in Figure 5.

During treatment, no adverse events were reported. The patient tolerated the combination therapy very well, both clinically and biologically, as expected.

## 3. Discussions

It is important to emphasize that the optimal multimodal treatment for GNET and CCS should be tailored to the individual patient, considering tumour biology, disease extent, overall health status, and the most current clinical evidence [3].

Surgical management remains the cornerstone of treatment for GNET and CCS, primarily due to the rarity of these tumours and the lack of robust clinical trial data. Surgery is often combined with adjuvant therapies, selected on a case-by-case basis. In this patient’s case, initial surgical intervention resulted in complete tumour resection and effective local control, achieving a PFS of approximately three years in the absence of systemic adjuvant treatment.

Although adjuvant treatment is often considered following surgery, GNET/CCS is generally regarded as poorly responsive to both radiotherapy and conventional chemotherapy, with conflicting evidence regarding any clear survival benefit. Clinical practice guidelines from ESMO-EURACAN-GENTURIS recommend individualized risk assessment—based on histological subtype, tumour grade, size, and location—along with a tailored follow-up strategy aimed at early detection of local or distant recurrence [10]. The most frequently used chemotherapy regimens for GNET/CCS include agents such as doxorubicin, sunitinib, and gemcitabine. However, their effectiveness remains limited. In a retrospective analysis, Smrke et al. emphasized that systemic therapies typically used for soft tissue sarcomas provide minimal clinical benefit in advanced CCS, with overall poor response rates [16]. Limited data, including findings by Nakai T. et al., suggest that trabectedin may offer potential antitumor activity in the treatment of GNET/CCS [22]. Regarding adjuvant treatment, one of the most frequently cited cases is the report by Singh et al., which described a patient with GNET who achieved a 7-year disease-free survival following adjuvant chemotherapy with a cisplatin and etoposide-based regimen [23]. In the case presented in this paper, several prognostic factors were considered, including tumour size, location, and histological grade. Based on these, we concluded that complete surgical resection was sufficient at the initial stage, and active surveillance was appropriate—resulting in a progression-free interval of three years.

In the metastatic setting, several retrospective analyses have explored the use of targeted therapies aimed at molecular alterations or signalling pathways implicated in GNET. These studies sought to identify potential druggable targets and assess the clinical efficacy of agents such as tyrosine kinase inhibitors (TKIs) and immunotherapies. Case-based and retrospective data evaluating targeted therapies—including MET inhibitors (such as tivantinib and crizotinib) and anti-VEGF agents (such as sunitinib, pazopanib, and cabozantinib)—have demonstrated limited and variable antitumor activity. Notably, Subbiah et al. reported the first documented case of GNET harbouring an EWSR1-CREB1 fusion, in which the patient achieved a significant and durable clinical response to combination therapy with the receptor tyrosine kinase inhibitors crizotinib and pazopanib, maintaining clinical benefit for over 1.5 years [24]. Furthermore, extrapolating from a Phase II EORTC study investigating crizotinib monotherapy in patients with MET-driven clear cell sarcoma CCS, which demonstrated stable disease in 7 out of 12 cases for approximately five months, targeted therapy may represent a more effective first-line option than conventional chemotherapy in select cases of GNET/CCS [24]. The EWSR1-CREB1 fusion is believed to upregulate the MET signalling pathway through the activation of microphthalmia-associated transcription factor (MITF), potentially creating a therapeutic vulnerability to MET inhibitors [25]. Data published by Chang et al. reported that apatinib and anlotinib may be effective treatment options for advanced gastrointestinal neuroectodermal tumours (GNETs), with the potential to prolong patient survival [12]. Additionally, Su et al., in a case report analysis, described a young patient who received fifth-line treatment with apatinib mesylate and sixth-line therapy with a combination of apatinib and temozolomide. Both apatinib-containing regimens achieved stable disease, with progression-free survival durations of 4.7 months for apatinib monotherapy and 3.1 months for the combination with temozolomide [26]. Stacchiotti S et al. reported on a tumour response to sunitinib malate (SM) in a 46-year-old female patient with metastatic clear-cell sarcoma [27]. Stacchiotti et al. reported a tumour response to sunitinib malate (SM) in a 46-year-old female patient with metastatic clear cell sarcoma, highlighting the potential efficacy of anti-angiogenic therapy in select cases [28]. Overall, treatment responses have generally been poor—typically partial and short-lived—with only limited and temporary disease stabilization observed in most cases.

Immunotherapy has shown promising results in the treatment of advanced sarcomas and may hold potential for GNET/CCS. A subgroup analysis from a non-randomized, open-label, phase II basket trial published by Blay et al. in 2023 [17] reported encouraging activity and manageable toxicity of pembrolizumab in several rare and ultra-rare sarcoma subtypes. These findings support the PD-1/PD-L1 axis as a potential therapeutic target in selected sarcoma histology [17]. Marcom et al. presented a case of recurrent, unresectable CCS that achieved a complete clinical response following treatment with pembrolizumab combined with radiotherapy, suggesting a potential synergistic effect between PD-1 blockade and radiation therapy [29]. Data from the SAINT phase I/II clinical trial, conducted by Gordon et al., demonstrated that the combined use of immune checkpoint inhibitors—ipilimumab and nivolumab—with the antineoplastic agent trabectedin significantly improved survival outcomes in patients with advanced sarcoma. Notably, clear cell sarcoma was among the subtypes showing a favourable response to this combination therapy [30].

The combination of targeted therapy and immunotherapy to achieve synergistic effects is already a standard approach in the treatment of several cancers, particularly lung and kidney tumours. In the context of GNET/CCS, the EWSR1-ATF1 translocation results in the replacement of the kinase-regulated domain of ATF1 with the N-terminal portion of EWSR1, leading to aberrant activation of the microphthalmia-associated transcription factor (MITF). MITF, in turn, plays a key role in oncogenesis by upregulating the c-MET gene, which encodes a receptor tyrosine kinase normally expressed on stromal and mesenchymal cells. This fusion-driven pathway is critical for tumour growth, as EWSR1-ATF1 is essential for c-MET expression, and malignant proliferation in these tumours appears to be dependent on c-MET signalling [31]. Cabozantinib is an oral small-molecule inhibitor that targets multiple tyrosine kinases, including MET and VEGFR2. It is currently approved for the treatment of metastatic renal cell carcinoma and metastatic medullary thyroid cancer. Emerging data evaluating cabozantinib in the treatment of soft tissue sarcomas have shown promising results, demonstrating both good tolerability and effective disease stabilization in a subset of patients [32]. Positive outcomes have been reported when cabozantinib is combined with immune checkpoint inhibitors, suggesting a potential synergistic effect that may enhance antitumor activity [32,33]. Cabozantinib was evaluated in combination with nivolumab and ipilimumab in a randomized phase II study comparing this triple combination to cabozantinib monotherapy. A total of 69 patients were randomized to the combination arm, while 36 received cabozantinib alone. The combination group demonstrated seven objective responses (11%), compared to two (6%) in the monotherapy arm. The median PFS was 5.3 months (95% CI, 4.1–11) for the combination therapy versus 3.5 months (95% CI, 1.1–7.7) for monotherapy (*p* = 0.016). The median overall survival (OS) was 22.6 months (95% CI, 14.8–not available) in the combination arm, while it was not reached in the monotherapy arm (95% CI, 9.6–not available), with a *p*-value of 0.42, indicating no statistically significant OS difference. Notably, among the 19 patients from the monotherapy arm who crossed over to the combination treatment, seven experienced tumour shrinkage—suggesting at least an additive, if not synergistic, effect of combining cabozantinib with nivolumab and ipilimumab [34]. Abstract 1725MO, presented at ESMO 2024, reported results from the clear cell sarcoma cohort of the IMMUNOSARC II master trial, a phase II study conducted by GEIS, ISG, and UCL evaluating the combination of sunitinib and nivolumab. At the time of analysis, 20 patients with EWSR1 rearrangements were evaluable for the primary endpoint. Among them, 28% remained on treatment, while 70% had discontinued therapy due to disease progression or toxicity. With a median follow-up of 12.6 months (95% CI: 1.10–24.10), the 6-month PFS rate was 50% (95% CI: 26–75), and the median PFS was 7.83 months (95% CI: 1.28–14.39). The median overall survival (OS) was 17 months (95% CI: 8–26), suggesting a clinically meaningful signal of activity for the sunitinib and nivolumab combination in patients with advanced clear cell sarcoma [18]. No further analyses were conducted, although disease stability lasted less than six months in most cases. However, the combination of targeted therapy and immunotherapy is a rapidly expanding approach. Case-based evidence from the literature suggests potential benefits in adding anti–PD-1/PD-L1 checkpoint inhibitors—aimed at reversing T-cell exhaustion—while synergizing with the effects of targeted therapies.

Rakefet S. et al. reported a robust clinical response in a patient with metastatic clear cell sarcoma treated with a combination of cabozantinib, nivolumab, and hapten dinitrophenyl-modified autologous tumour cells. The patient achieved a partial response and remained progression-free for two years, underscoring the potential of such combinatorial strategies in select cases [19]. A single-arm, phase Ib/II clinical trial enrolled adult patients with selected subtypes of soft tissue sarcoma. A total of 68 patients were enrolled 16 in the phase Ib portion and 52 in phase II. The recommended phase II dose of sunitinib was 37.5 mg during the induction phase, followed by 25 mg when combined with nivolumab. After a median follow-up of 17 months (range: 4–26 months), the 6-month PFS rate was 48% (95% CI: 41–55%). The most common grade 3–4 adverse events were transaminitis (17.3%) and neutropenia (11.5%). The trial concluded that the combination of sunitinib and nivolumab demonstrated clinical activity with manageable toxicity in patients with advanced soft tissue sarcoma, with nearly half of the patients remaining progression-free at 6 months [35].

For our patient with EWSR1/CREB1 fusion-positive GNET, we selected a combination of immunotherapy and targeted therapy following disease relapse with hepatic and peritoneal metastases. This decision was based on the potential synergistic effect of combining PD-1 blockade with cabozantinib—an agent with a well-established and manageable toxicity profile. The use of NGS and molecular diagnostics played a pivotal role in guiding personalized treatment decisions.

First-line treatment options were evaluated through case-based data in the literature [19,20]. Based on these findings, we selected cabozantinib as a potentially effective and life-prolonging therapy, paired with immunotherapy to address T-cell exhaustion and enhance antitumor activity. This approach aimed to maximize efficacy while minimizing adverse events.

Therapeutic selection was guided by molecular profiling and tumour characteristics, particularly the NGS results from liver and peritoneal metastases, which revealed: PD-L1 expression of 15%, presence of the EWSR1/CREB1 fusion, no other actionable oncogenic drivers, and a Ki-67 index of 30%. Cabozantinib was specifically recommended based on the NGS report. While sunitinib or pazopanib were considered as secondary options, their higher toxicity profiles made them less favourable. Chemotherapy was declined by the patient due to concerns over cytotoxic side effects and their impact on quality of life. In contrast to previously reported cases treated with tyrosine kinase inhibitors alone, our patient has maintained a longer period of stable disease without toxicity, suggesting that immunotherapy may have contributed additional benefit. Differences in molecular fusion type (EWSR1/CREB1 versus EWSR1/ATF1), tumour burden at relapse, and prior treatments may partly explain the variability in responses across cases. Further accumulation of such clinical experiences will be critical to delineate predictive factors for response.

Importantly, the known safety profile of the cabozantinib–nivolumab combination presented no management concerns. This is supported by findings from the CheckMate 9ER trial, which demonstrated the superiority of cabozantinib plus nivolumab over sunitinib in previously untreated advanced renal cell carcinoma (RCC), showing improved progression-free survival, overall survival, and objective response rates. Toxicities were manageable with supportive care, dose modifications for cabozantinib, and standard immunosuppressive strategies for nivolumab [36]. In our patient’s case, treatment was well tolerated with no reported adverse events.

## 4. Conclusions

It is essential that treatment decisions for patients with GNET and CCS be made within a multidisciplinary team setting, involving surgical oncologists, radiation oncologists, medical oncologists, pathologists, and molecular diagnostics experts—ideally within dedicated cancer centres that specialize in rare tumours. By leveraging advancements in research, technology, and molecular understanding of tumour biology—particularly through NGS—clinicians can offer tailored therapies that improve outcomes and enhance patient care in these ultra-rare malignancies.

In our EWSR1/CREB1 fusion-positive GNET case, immunotherapy combined with targeted therapy was chosen as the rational and promising therapeutic option at the time of relapse, following a three-year disease-free interval after initial surgical management, which warrants further investigation. The patient presented with hepatic and peritoneal metastases, and the rationale for treatment was based on the potential synergistic effect of combining nivolumab with cabozantinib. Over one year after initiating therapy, the patient has maintained stable disease for 18 months and experienced no treatment-related adverse events, highlighting the benefit of a personalized, molecularly guided approach.

This single case underscores the critical role of NGS and molecular diagnostics in enabling targeted therapies for rare solid tumours. It also supports the rationale for recommending tyrosine kinase inhibitors, such as cabozantinib, based on molecular profiling, and suggests that combining such agents with immunotherapy may be a valid therapeutic strategy—particularly when conventional options are lacking. Finally, ethical and access considerations must be acknowledged. In Romania, only chemotherapy is reimbursed for rare sarcomas, while targeted therapy and immunotherapy are not covered, requiring patients to self-fund treatment. This inequity highlights the challenges of applying precision oncology in real-world settings and underscores the importance of healthcare policy in ensuring equitable access to innovative therapies.

Although immunotherapy and targeted therapies represent promising avenues in GNET/CCS management, there are currently no established clinical standards. Most available evidence is derived from case reports and small retrospective series. This highlights the importance of publishing each clinical experience to build a more robust evidence base. Reporting on therapeutic strategies for such diseases, where clinical guidelines are lacking, is of great value to the oncology community. Prospective clinical trials in this setting are difficult to conduct due to the rarity of the disease, and therefore, treatment approaches may need to be extrapolated from data on soft tissue sarcomas. Continued collaboration, case documentation, and translational research are essential to optimizing personalized and multidisciplinary treatment strategies in this rare pathology. Future research should aim to systematically evaluate the efficacy and safety of combined immunotherapy and targeted therapy in GNET and CCS through collaborative registries and multicentre consortia, given the challenges of conducting large-scale clinical trials in ultra-rare malignancies. Furthermore, functional studies exploring the biological consequences of EWSR1-CREB1 and related fusions may reveal novel therapeutic vulnerabilities. Expanding the use of comprehensive genomic profiling across cases will not only facilitate individualized treatment but also contribute to identifying molecular subgroups that could benefit from specific targeted or immune-based interventions. Ultimately, integrating molecular insights with prospective data collection may pave the way for establishing evidence-informed treatment guidelines in these rare tumour entities.

## Figures and Tables

**Table 1:** Immunohistochemical staining results sustaining the diagnosis.

Date	Institution	Tests	Results	Interpretation
11 June 2020	Institute of Oncology Bucharest, Bucharest, Romania	S100	diffuse positive	The differential diagnosis is between a gastrointestinal clear cell sarcoma and a melanoma metastasis; it is recommended to complete with additional melanoma markers (MITF, CK 19, SOX10 for confirmation and possibly to complete the diagnosis within situ hybridization for EWSR1-CERB1 or with PCR techniques
		CD56	zonal positive	
		Synapto	zonal positive	
		EMA	membrane expression in restricted areas	
		AE 1/AE3, CK 20, CK7, CDX2, HMB45, MART, DOG1, CD 117, CD 34, Desm	Negative	
		VIM	positive in restricted areas	
		Ki67	35%	
29 June 2020	“Victor Babes” Institute, Bucharest, Romania	S100, SOX10	Positive	The histological appearance and the immunohistochemical profile support the diagnostic of GNET/CCS)
		SMA, CD34, CD21, DOG1, Chromogranin, PAX8	Negative	
27 July 2020	Medcenter, Bucharest, Romania	pS100, Synaptophysin, CD99	diffuse positive	The histological appearance and the immunohistochemical profile support the previous diagnostic of GNET; the EWSR1 gene translocation panel (ATF or CREB1) is recommended
		SOX10	diffuse positive	
		Pan Melanoma cocktail, CK 19	Negative	
16 September 2020	MD Anderson Cancer Center, Madrid, Spain	Vimentin, CD199, S100, SOX10, CD56, Synaptophysin	Positive	Histology/immunohistochemical and molecular findings (EWSR1-CREB1 fusion present) are consistent with malignant GNET
		HMB45, MART-1, cytokeratin, CDX-2, CKIT/CD 117, DOG-1, CD 34, Actin, Desmin	Negative	

**Table 2:** NGS (Foundation One) CDx results.

Genomic Signatures	Result	Actionability
Microsatellite status (MS)	MS-stable	No therapies or clinical trials
Tumour Mutational Burden (TMB)	4 muts/Mb	No therapies or clinical trials
PDL1	Negative	
MYC-A59V alteration	Transcript number: NM_002467, Coding sequence effect 176C > T)	No therapies approved; no trials available
EWSR1	EWSR1-CREB1 fusion	No therapies approved; no trials available
Gene STAG 2	Alteration: R1045, Transcript number NM_006603, Coding sequence effect 3133C > T 30	No therapies approved; no trials available

NOTE: One or more variants of unknown significance (VUS) were detected in this patient’s tumour. These variants may not have been adequately characterized in the scientific literature at the time this report was issued, and/or the genomic context of these alterations makes their significance unclear. They were included hen in the event that they become clinically meaningful in the future. BCOR-V679I; CDK12-P1257del, CREBBP-S128C; ERBB3 rearrangement, IRS2-K1170R; KDM5C-R1435C; MLL2-L4077F; SMO-Q745R.

## Data Availability

The original contributions presented in this study are included in the article. Further inquiries can be directed to the corresponding authors.
